# Long-term management of aortic stenosis (surgery and transcatheter options in young patients): a case report of valve-in-valve transcatheter aortic valve implantation

**DOI:** 10.1093/ehjcr/ytaf638

**Published:** 2025-12-12

**Authors:** Giulio Russo, Marcello Marchetta, Aris Moschovitis, Giuseppe Massimo Sangiorgi, Maurizio Taramasso

**Affiliations:** Department of Biomedicine and Prevention, Cardiology Unit, Policlinico Tor Vergata, University of Rome, 00100 Rome, Italy; Department of Biomedicine and Prevention, Cardiology Unit, Policlinico Tor Vergata, University of Rome, 00100 Rome, Italy; HerzZentrum Hirslanden, 8008 Zurich, Switzerland; Department of Biomedicine and Prevention, Cardiology Unit, Policlinico Tor Vergata, University of Rome, 00100 Rome, Italy; HerzZentrum Hirslanden, 8008 Zurich, Switzerland

**Keywords:** Case report, Valve-in-valve, Transcatheter aortic valve implantation, Valve degeneration, Aortic regurgitation, Structural valve deterioration, Lifetime management

## Abstract

**Background:**

Recent evidences have supported the use of transcatheter aortic valve implantation (TAVI) across all risk categories of patients. However, in case of young patients several points should be considered in a lifetime perspective taking into account both surgical and percutaneous therapies.

**Case summary:**

An 82-year-old man with a history of aortic valve disease treated 12 years earlier with surgical aortic valve and aortic root replacement was admitted for worsening dyspnoea. Echocardiogram showed degenerated surgical bioprosthesis with severe regurgitation. Due to prior surgery and high operative risk, the heart team opted for valve-in-valve TAVI. The procedure was successful and the patient remained asymptomatic at 30-day follow-up.

**Discussion:**

This case shows how to approach aortic valve disease in a lifetime perspective. Surgical and percutaneous approaches can be used in multiple combinations according to patient’s age, anatomical characteristics and personal preferences.

Learning points

- Lifetime management perspective is fundamental in young patients affected by aortic valve disease.
- When surgery is selected as first procedure, it should take into account possible need for a second surgical/percutaneous intervention and, consequently, some technical preventive measures should be considered (e.g. coronary ostia re-implantation and prosthesis sizing).

## Introduction

Transcatheter aortic valve implantation (TAVI) is now indicated in all risk categories of patients with severe aortic stenosis and is considered as the first-line therapy for patients ≥75 years.^1,2^ However, while approaching patients with lower procedural risk also the median age tends to decrease although outcomes of TAVI in young patients are scarce. On this ground, heart team discussion has shifted towards lifetime management of aortic stenosis taking into account both surgical and percutaneous therapies and their possible combinations in case of possible second or even third re-intervention.^3^ In this perspective, it is crucial a careful assessment and selection of the first intervention in order to allow future re-interventions.

## Summary figure

**2012**

Surgical aortic root replacement with biological composite graft and re-implantation of coronary ostia

**2012-24**

Stable clinical course without symptoms

**Early 2025**

Progressive exertional dyspnoea (NYHA III)

**March-April 2025**

Echocardiography confirms bioprosthetic valve degeneration with severe aortic regurgitation

**May 2025**

Valve-in-valve TAVI

**May 2025 (day 7)**

Discharged in stable condition

**June 2025**

30-Day follow-up: asymptomatic, normal valve function on echocardiogram

## Case presentation

An 82 years old man was admitted to our institution with progressive exertional dyspnoea (New York Heart Association, NYHA, functional class III). His past medical history was significant for surgical aortic root and valve replacement with a bioprosthesis 12 years earlier and atrial fibrillation. For over a decade, the patient remained in excellent clinical condition, with regular clinical and echocardiographic follow-up.

Over the last months, the patient started to complain about worsening fatigue and dyspnoea during daily activities. On clinical examination, the patient had high pulse pressure while auscultation revealed a diastolic murmur over the aortic area. Electrocardiogram showed atrial fibrillation with good rate control.

Transthoracic echocardiography revealed severe regurgitation of the surgical aortic bioprosthesis, with mildly reduced left ventricular ejection fraction (LVEF = 45%), mild to moderate mitral regurgitation and moderate tricuspid regurgitation (see Supplementary material online, *Video S1*). These findings were confirmed on transesophageal echocardiography, which also excluded endocarditis or thrombus (see Supplementary material online, *Video S2*).

The patient was referred to the local heart team for multi-disciplinary evaluation. Due to his age, previous surgical interventions and high operative risk (STS Score >8%, TRIVALVE Score = 3, TRISCORE = 7), he was considered unsuitable for redo surgery and therefore selected for percutaneous intervention.^4,5^

**Figure 1:**
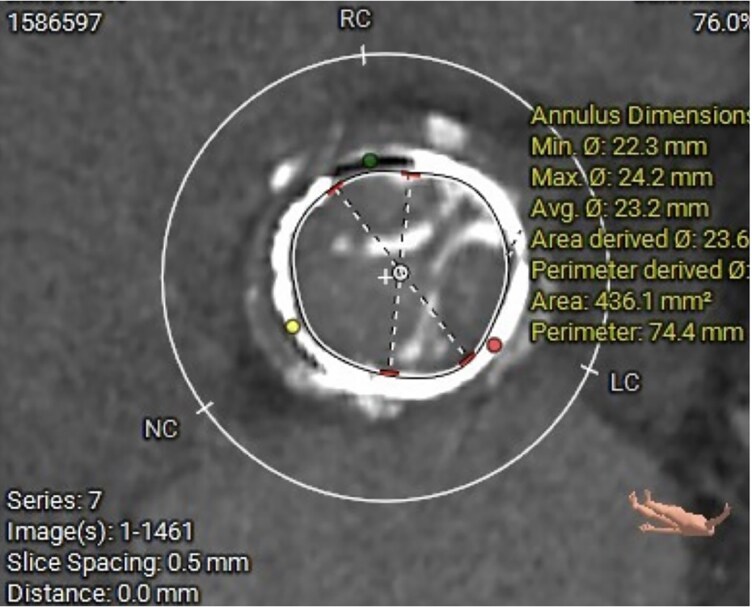
Pre-procedural CT assessment of the aortic root and bioprosthesis.

**Figure 2:**
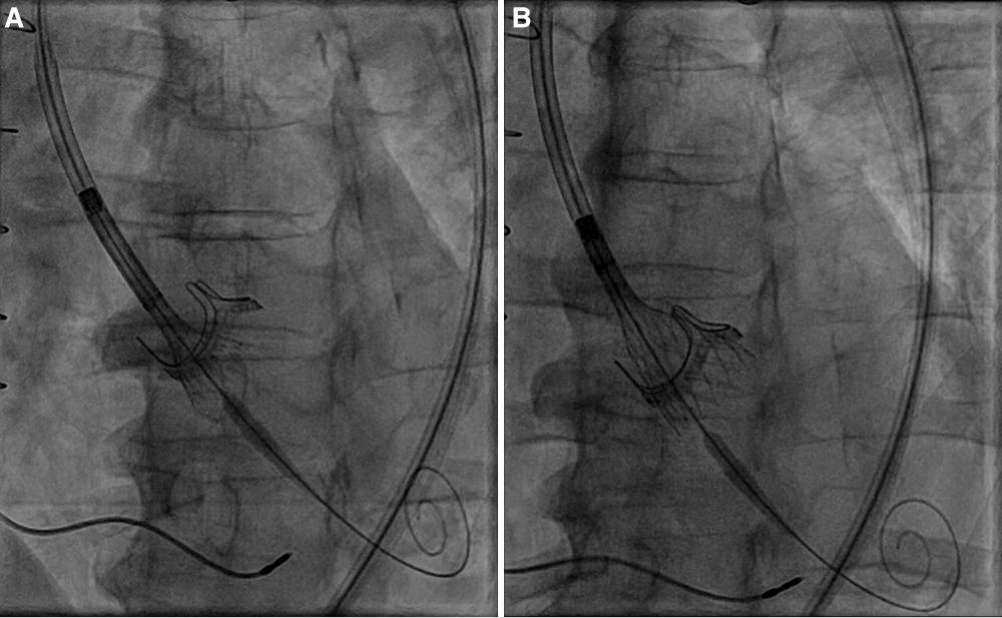
Fluoroscopic view during valve-in-valve TAVI deployment (*A* and *B*).

**Figure 3:**
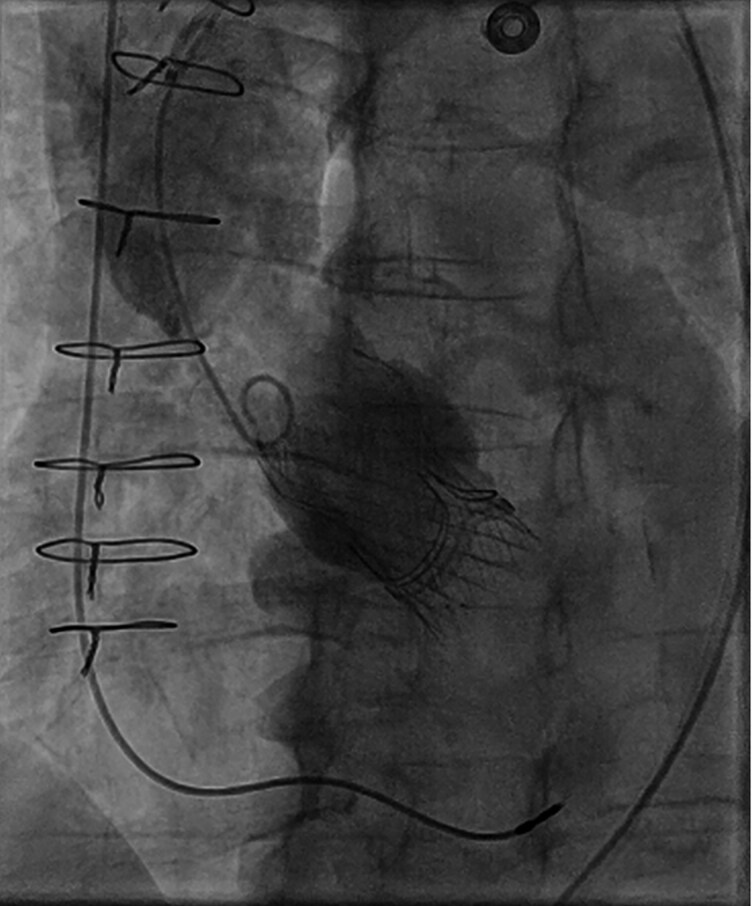
Post-deployment aortography.

Computed tomography (CT) scan was performed for ViV-TAVI procedural planning: Annulus perimeter was 74 mm, ascending aorta diameter of 42 mm, right and left coronary height from the valvular plane of 18 and 16 mm, respectively (*Figure 1*). The patient underwent transfemoral ViV-TAVI with implantation of a 29 mm Evolut FX valve (Medtronic, US) (*Figures 2* and *3*). Fluoroscopic and echocardiographic imaging confirmed optimal valve positioning and function, with a post-implant mean gradient of 8 mmHg and no paravalvular leak.

The patient was discharged on day 7 and at 30-day follow-up; he was asymptomatic for dyspnoea, with echocardiographic evidence of a well-functioning transcatheter aortic valve.

## Discussion

The recent evidences from randomized controlled trials on low-risk patients undergoing TAVI for severe aortic stenosis have brought to the approval of TAVI also in lower risk categories of patients, which, in some cases, overlaps with young patients. However, data on the use of TAVI in young patients are scarce and surgery remains the first-line option. However, whatever the treatment selected, either surgical or percutaneous, it is fundamental in young patients to approach the disease in a lifetime perspective, taking into account possible re-interventions. Consequently, when selecting the first treatment, it is important to consider not only the age, but also technical and anatomical patient’s characteristics in order to facilitate and not to preclude possible future re-interventions. Several key outstanding issues should be carefully weighted between surgery and transcatheter aortic valve prosthesis:

- Paravalvular leak
- Conduction disturbances and pacemaker need
- Prosthesis-patient mismatch
- Coronary access
- Prosthesis durability and re-intervention

In the case presented, the patient had undergone aortic surgery at the age of 70 due to bicuspid aortic valve complicated by aortic aneurism and aortic insufficiency. At this stage of the aortic disease, surgery was the indicated treatment due to the combination of ascending aorta aneurism and aortic valve regurgitation. In such intervention, a Bio-Bentall (27 mm) procedure with neo-Valsalva and native coronary arteries re-implantation was performed. Some key technical aspects are noteworthy for the lifetime management of this case: (i) high native coronaries re-implantation from the valvular plane in order to facilitate possible future re-interventions and (ii) the selection of a bioprosthesis, which keeps open the TAVI-in-SAVR option and finally (iii) the neo-Valsalva as it improves valve performance and, possibly, increases valve durability. The treatment strategy with biological prostheses was based on current guidelines (both American and European) and on some evidences showing similar mortality between surgical biological and mechanical valves in patients 50–69 years of age and suggesting no additional survival benefit in mechanical compared to biological valves in patients >55 years.^6,7^ As expected for bioprosthesis, after 12 years it degenerated with severe aortic regurgitation occurrence. In consideration of the previous surgical intervention and the comorbidities, the patient was deemed at high risk for redo surgery and a percutaneous approach with ViV TAVI was selected. In particular, the supra-annular design of the Evolut FX (Medtronic, US) could reduce the mean gradient across the valve and the possible risk of patient-prosthesis mismatch. At the same time, the new FX design combined with commissural alignment could facilitate coronary re-access, although this concept might be less reliable for TAVI-in-SAVR.

This case highlights how prior surgical planning can facilitate future transcatheter interventions in the context of aortic valve disease. Similar cases with young patients affected by aortic valve disease are increasing and the need for a lifetime perspective approach is becoming fundamental and, while technologies, techniques (surgical and transcatheter) and robotics are developing and improving, the same approach might be applied to all heart valve disease.^8–11^

## Conclusion

This case illustrates the importance of lifetime management of aortic valve disease in young patients. First, the patient underwent surgical Bio-Bentall intervention and after valve degeneration, 12 years later, a ViV TAVI was performed with good technical and clinical results.

## Lead author biography


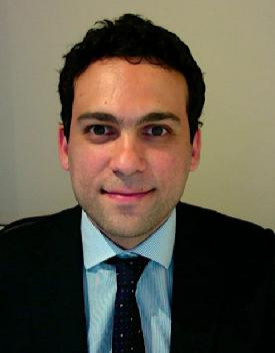


Giulio Russo is an interventional cardiologist at Policlinico Tor Vergata University Hospital in Rome with specific interest in complex coronary interventions and structural heart disease interventions.

## Supplementary Material

ytaf638_Supplementary_Data

## Data Availability

Data are available upon reasonable request.
